# An Assembly Model of Rift Valley Fever Virus

**DOI:** 10.3389/fmicb.2012.00254

**Published:** 2012-07-19

**Authors:** Mirabela Rusu, Richard Bonneau, Michael R. Holbrook, Stanley J. Watowich, Stefan Birmanns, Willy Wriggers, Alexander N. Freiberg

**Affiliations:** ^1^School of Biomedical Informatics, University of Texas Health Science Center at HoustonHouston, TX, USA; ^2^Center for Genomics and Systems Biology, Biology Department, New York UniversityNew York, NY, USA; ^3^Computer Science Department, Courant Institute of Mathematical Sciences, New York UniversityNew York, NY, USA; ^4^Department of Pathology, Institute for Human Infections and Immunity, University of Texas Medical BranchGalveston, TX, USA; ^5^National Institute of Allergy and Infectious Diseases-Integrated Research FacilityFrederick, MD, USA; ^6^Department of Biochemistry and Molecular Biology, University of Texas Medical BranchGalveston, TX, USA; ^7^Department of Physiology and Biophysics, Institute for Computational Biomedicine, Weill Medical College of Cornell UniversityNew York, NY, USA

**Keywords:** bunyavirus assembly, protein structure prediction, hybrid modeling, multi-body refinement, multi-resolution registration

## Abstract

Rift Valley fever virus (RVFV) is a bunyavirus endemic to Africa and the Arabian Peninsula that infects humans and livestock. The virus encodes two glycoproteins, Gn and Gc, which represent the major structural antigens and are responsible for host cell receptor binding and fusion. Both glycoproteins are organized on the virus surface as cylindrical hollow spikes that cluster into distinct capsomers with the overall assembly exhibiting an icosahedral symmetry. Currently, no experimental three-dimensional structure for any entire bunyavirus glycoprotein is available. Using fold recognition, we generated molecular models for both RVFV glycoproteins and found significant structural matches between the RVFV Gn protein and the influenza virus hemagglutinin protein and a separate match between RVFV Gc protein and Sindbis virus envelope protein E1. Using these models, the potential interaction and arrangement of both glycoproteins in the RVFV particle was analyzed, by modeling their placement within the cryo-electron microscopy density map of RVFV. We identified four possible arrangements of the glycoproteins in the virion envelope. Each assembly model proposes that the ectodomain of Gn forms the majority of the protruding capsomer and that Gc is involved in formation of the capsomer base. Furthermore, Gc is suggested to facilitate intercapsomer connections. The proposed arrangement of the two glycoproteins on the RVFV surface is similar to that described for the alphavirus E1-E2 proteins. Our models will provide guidance to better understand the assembly process of phleboviruses and such structural studies can also contribute to the design of targeted antivirals.

## Introduction

Rift Valley fever virus (RVFV) is a member of the family *Bunyaviridae* (genus *Phlebovirus*), transmitted primarily by mosquitoes and is endemic throughout much of Africa and, in recent years in the Arabian Peninsula. The virus causes outbreaks in a wide range of vertebrate hosts, with humans and livestock being the most affected. Infection of livestock can result in economically disastrous abortion storms and high mortality among young animals. In humans, the virus causes a variety of pathologic effects with less than 1% of infections thought to result in fatal hemorrhagic fever or encephalitis (MMWR, 2007). However, during the outbreak in Kenya, from November 2006 to January 2007, the fatality rate in humans reached nearly 30% (MMWR, 2007). RVFV is considered a high consequence emerging infectious disease threat and is also of concern as a bioterrorism agent. RVFV is classified as Category A select agent by CDC and USDA. Currently, there are no commercially available vaccines or therapeutics.

RVFV is a typical enveloped bunyavirus and has a tri-segmented, negative-sense RNA genome, and most likely enters the host cells via receptor-mediated endocytosis, which requires an acid-activated membrane fusion step (Lozach et al., 2010, 2011). The two glycoproteins, Gn and Gc, are expressed as a precursor polypeptide, which is then co-translationally cleaved prior to maturation of the envelope glycoproteins (Collett et al., 1985; Wasmoen et al., 1988). For transport from the endoplasmic reticulum to the Golgi apparatus, both newly synthesized glycoproteins are required (Gerrard and Nichol, 2002). Within the virion, the surface glycoproteins are anchored in the envelope membrane as type-I integral membrane proteins and are responsible for receptor recognition and binding, and entry into target cells through fusion between viral and cellular membranes. In contrast to most other negative-stranded RNA viruses, bunyaviruses lack a matrix protein and the cytoplasmic tails of Gn and Gc likely interact directly with the ribonucleoprotein complex inside the virus particle (Overby et al., 2007; Piper et al., 2011). Gn and Gc form oligomers and are organized on the virus surface as cylindrical hollow spikes that cluster into distinct capsomers. The virus surface is covered with 122 capsomers arranged on an icosahedral lattice with a triangulation number of 12 (Freiberg et al., 2008; Huiskonen et al., 2009; Sherman et al., 2009). Computational studies have predicted RVFV Gc to be a class II viral fusion protein (Garry and Garry, 2004). Owing to their importance in the process of virion maturation, receptor binding, and fusion with the host cell, both glycoproteins form attractive targets for the design of antiviral drugs blocking the receptor binding and/or fusion processes.

Structural data for bunyavirus glycoproteins are available for the hantavirus and Crimean–Congo hemorrhagic fever virus Gn cytoplasmic tails (Estrada et al., 2009, 2011; Estrada and De Guzman, 2011). However, no crystallographic data are available for any bunyavirus glycoprotein ectodomain. Bioinformatic investigation and molecular homology modeling of the bunyavirus Gc proteins of the five different genera revealed that they share a limited number of similar sequences with each other and that they have sequence similarity with the alphavirus E1 protein, suggesting that bunyavirus Gc proteins could be class II viral fusion proteins (Garry and Garry, 2004; Tischler et al., 2005; Plassmeyer et al., 2007; Hepojoki et al., 2010). Further, experiments with members from other bunyavirus genera supported the major role Gc plays during fusion with the host cell membrane and entry (Plassmeyer et al., 2005, 2007; Shi et al., 2009). Three-dimensional (3D) molecular model structures for Gc have been described for members of different genera, such as La Crosse virus (*Orthobunyavirus*), Sandfly fever virus (*Phlebovirus*), Andes and Tula viruses (Hantaviruses), and have been used successfully to study the functionality of fusion peptides and the interaction and oligomerization of glycoproteins (Garry and Garry, 2004; Tischler et al., 2005; Hepojoki et al., 2010; Soldan et al., 2010). Most of these studies targeted the Gc protein; much less information is available for the Gn protein. It has been suggested that the phlebovirus Gn plays a role in receptor binding and that it might have structural similarity to the alphavirus E2 protein (Garry and Garry, 2004).

To better understand the assembly of bunyaviruses and the functional interaction between Gn and Gc glycoproteins, we sought to generate 3D structure models for RVFV Gn and Gc monomers using bioinformatic approaches. Specifically, homology models were created following established virus protein prediction strategies (Garry and Garry, 2004, 2008, 2009; Tischler et al., 2005; Lee et al., 2009; Hepojoki et al., 2010). Subsequently, we used these model structures to evaluate possible positions within the existing cryo-electron microscopy (cryoEM) density map of RVFV virions to predict protein–protein interaction interfaces and to propose an assembly model for RVFV. We suggest that RVFV Gn and Gc are arranged topologically within the virus particle, with some similarity to the E1 and E2 proteins of alphaviruses. Our model indicates that RVFV Gn could be involved in receptor binding and covers the fusion loop of Gc at neutral pH, while Gc is proposed to play a major role during the membrane fusion step.

## Materials and Methods

### Protein sequence analysis

For sequence and structural analysis, the RVFV vaccine strain MP-12 glycoprotein encoding nucleotide sequence (GenBank DQ380208) was used. The secondary structure of RVFV Gn and Gc, respectively, were examined using Jpred3^1^ (Cole et al., 2008). To define the location of the glycoprotein transmembrane domains (TMD) (Table 1), as well as cytoplasmic tail domains (CTD), the EXPASY^2^, HMMTOP^3^, SOSUI^4^, and TMHMM^5^ servers were used (Hirokawa et al., 1998; Tusnady and Simon, 1998; Krogh et al., 2001). We used the NetN Glyc 1.0 Server^6^ to predict the locations of N-glycosylation sites.

**Table 1:** **Prediction of location of transmembrane domains**.

	RVFV Gn^a^	RVFV Gc^a^
EXPASY	429–449 [21]^b^	470–490 [21]
HMMTOP	429–451 [23]	470–494 [25]
	517–535 [19]^c^
SOSUI	433–455 [23]	469–491 [23]
	515–536 [22]^c^
TMHMM	432–454 [23]	469–491 [23]
Average	429–455 [27]	469–494 [26]
	515–536 [22]^c^

*^a^RVFV MP-12 glycoprotein length [SwissProt #P21401] Gn: 536 aa; Gn: 507 aa*.

*^b^Numbers indicate the length of the transmembrane domains*.

*^c^Second TMD in Gn corresponds to signal peptide*.

### Protein structure prediction

Initial backbone models were generated using the fold recognition Meta Server^7^ (Kajan and Rychlewski, 2007), which used alignments from the FFAS_03 program^8^ to the two templates (Jaroszewski et al., 2005). These models agreed with alignments found using other fold recognition methods, increasing our confidence in these fold predictions. Side chains were added and models were refined using Modeller^9^ (Eswar et al., 2006). The atomic model of the Gn glycoprotein was generated based on the 1918 influenza H1 hemagglutinin protein (PDB ID: 1RD8, Stevens et al., 2004), specifically the HA1 chain, for which a 14.15% sequence identity was observed. Similarly, the atomic model of the Gc glycoprotein was built based on the Semliki Forest virus (SFV) structural E1 protein fitted into the Sindbis virus cryoEM map (PDB ID: 1LD4, Zhang et al., 2002) from an observed sequence identity of 13.83%. In addition to these structures sub-optimal FFAS_03 alignments and derived models were also evaluated in the context of the cryoEM density including alignments of RVFV Gc to PDB structures of the Chikungunya E1-E2 envelope glycoprotein complex fitted into the SFV cryoEM map (PDB ID: 2XFC; PDB ID: 1RER, Gibbons et al., 2004; Li et al., 2010), dengue virus E protein (PDB ID: 1P58, Zhang et al., 2003), integrin binding fragment of human fibrillin-1 (PDB ID: 1UZJ, Lee et al., 2004) and alignment of Gn to the EAP45/ESCRT GLUE domain (PDB ID: 2HTH–chain A, Alam et al., 2006). All of the proteins identified as similar to Gc are class II fusion proteins, and due to the similar sequence identity between the different homologs, an alternative atomic model of the RVFV Gc protein was built based on the structure of the Chikungunya virus E1 protein fitted into the cryoEM reconstruction of SFV (PDB ID: 2XFC; chain A, Voss et al., 2010). Since the overall shape of Gc is conserved between the models based on the Sindbis virus and SFV E1 protein, we did not further evaluate their positioning in the RVFV cryoEM map.

### Fitting of glycoprotein structures into the cryoEM density

The 3D models of the RVFV Gn and Gc glycoproteins were fitted into the RVFV vaccine strain MP-12 cryoEM map (Sherman et al., 2009). The organization of the two glycoproteins within the RVFV envelope was identified following a hybrid approach that combined an interactive exploration of the exhaustive search outcome with a multi-body refinement procedure. The multi-body refinement is described in detail in Birmanns et al. (2011) and a summary is provided here. The multi-step approach (Figure 2; see Results) was applied to generate an atomic model for the triangular face of the RVFV. First, an exhaustive search using the tool colores from the package Situs^10^ (Wriggers, 2010) was applied to explore possible placement for each of the Gc and Gn glycoproteins. The molecular modeling software Sculptor^11^ (Birmanns et al., 2011) was used for the interactive exploration of the exhaustive search results to select placements that are in agreement with computed Gn/Gc ratio within each capsomer type (Huiskonen et al., 2009; Sherman et al., 2009) and that show reduced steric clashing. Several such docking locations were identified for both Gc and Gn, and multiple models were iteratively refined by searching for the architecture that best described the density of the asymmetric unit.

## Results

### Fold recognition of the RVFV glycoproteins

**Figure 1:**
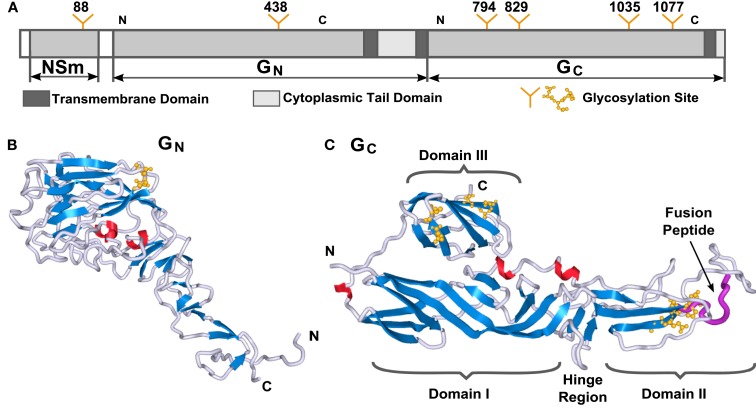
**Three-dimensional structure models of RVFV Gn and Gc proteins**. **(A)** Schematic representation of the RVFV M-segment polyprotein. Transmembrane and cytoplasmic tail domains are highlighted in dark gray or white bars, respectively. N-Glycosylation sites are indicated with the position of the respective Asn residue. The regions of the two glycoproteins used for molecular modeling are indicated with N and C. 3D molecular models for RVFV **(B)** Gn and **(C)** Gc are shown. Secondary structures are highlighted in blue for β-strands, red for α-helices, and gray for turns. The predicted location of the fusion peptide within Gc is represented in purple. The domain nomenclature in modeled Gc were used in adoption to the alphavirus E1 protein. The molecular graphics in this paper were generated with Sculptor (Birmanns et al., 2011) and Chimera (Pettersen et al., 2004).

Both RVFV glycoproteins, Gn and Gc, are known to be type-I integral transmembrane proteins. Before obtaining fold recognition and molecular model predictions of the two RVFV glycoproteins, the primary amino acid sequences of the entire Gn and Gc were analyzed for predicted TMD, ecto- and endo-domains (CTD), glycosylation sites, and consensus secondary structure prediction elements (Figure 1A). Gn is predicted to displays a mixture of α-helical, β-strands, and random coil secondary structural elements (Figure 1B). The N-terminus has a slightly higher content of β-strands, while the C-terminus is rich in α-helical elements located in the regions predicted for the TMD and CTD. Rift Valley fever virus Gc exhibits predominantly β-strands, a very low content of α-helices and a high content of random coiling (Figure 1C). Most of the α-helical elements are found in the regions predicted for the transmembrane and short CTD, as already described for the Gn protein. Garry and Garry (2004) suggested that the Gc glycoproteins of bunyaviruses are class II viral fusion proteins. Class II fusion proteins, such as the envelope glycoprotein E of tick-borne encephalitis virus and the E1 protein of Sindbis virus, are composed mostly of antiparallel β-sheets, similar to the secondary structure prediction for RVFV Gc.

### Model building and structural description of the RVFV Gn and Gc glycoproteins

To model the 3D structures of Gn and Gc, and to verify that RVFV Gc adopts a class II fusion protein fold, we initially focused on the near full-length RVFV Gn (530 aa in length) and Gc (507 aa in length) protein sequences (Figure 1A). However, molecular models could only be generated for the two glycoprotein ectodomains, so the TMDs and CTDs were removed from further analysis. Throughout the manuscript, the terms RVFV Gn and Gc are used to describe the ectodomain for each glycoprotein and not the entire glycoprotein itself.

The fold recognition revealed that the best matching profile for RVFV Gn resulted in a hit which had structural similarity to the Influenza 1918 human H1 hemagglutinin, specifically the receptor binding domain HA1 (Figure 1B). The molecular model generated for RVFV Gc was obtained based on the Sindbis virus and Chikungunya E1 proteins (Figure 1C). This result was expected, since bioinformatic analysis had already predicted that the bunyavirus Gc protein has sequence similarity with the alphavirus E1 protein, suggesting that bunyavirus Gc proteins are class II viral fusion proteins (Garry and Garry, 2004). Furthermore, all of the proteins identified as similar to Gc are class II fusion proteins (see Materials and Methods). As shown in Figure 1C, the modeled structure for RVFV Gc resembles the overall fold of a class II fusion protein (Kielian, 2006; Kielian and Rey, 2006).

The Gn and Gc model was evaluated in terms of stereochemical and geometric parameters such as bond lengths, bond angles, torsion angles, and packing environment and was found to satisfy all stereochemical criteria (assessed by VADAR statistics software package; Willard et al., 2003). For the 3D models, the (Φ, Ψ) values calculated for each amino acid residue of the individual model structures were within the allowed region of the Ramachandran plot (Ramachandran and Sasisekharan, 1968; data not shown).

The Gc protein consists of three domains, with predominantly β-strand content, which is in accordance with the amino acid sequence analysis (data not shown). The nomenclature of these three domains has been defined by analogy with the alphavirus E1 protein, domain I (central domain), domain II and domain III. Domain II contains two predicted glycosylation sites at positions N794 and N829 and also bears the predicted fusion loop of RVFV Gc that potentially inserts into the target host membrane during the pH-dependent virus fusion step (Garry and Garry, 2004). The location of the fusion loop is highlighted in purple in Figure 1C. Domain III, separated from the first two domains by a short stretch, forms an Ig-like β-barrel structure and contains two glycosylation sites at positions N1035 and N1077. On-going studies in our laboratory found that removal of the glycosylation sites in Gc has a negative effect on virus assembly and maturation (ANF, unpublished results). In contrast, the predicted 3D model for the ectodomain of RVFV Gn represents an elongated structure with a globular head domain (Figure 1B). The membrane-distal domain consists of a globular head, which displays a mixture of β-strands, and slightly less α-helical and random coil content. A stem-like region connects the globular domain with the TMD, which is not displayed in the 3D structure. The head domain also contains the glycosylation site at position N285.

The predicted N-glycosylation sites were in agreement with the findings from Kakach et al., 1989; yellow spheres in Figures 1B,C). All glycosylation sites on Gn and Gc are fully surface accessible, which supports our model structures.

### Glycoprotein modeling in the RVFV particle

Recently, we determined the 3D structure of the RVFV vaccine strain MP-12 by single-particle cryoEM at 27 Å resolution (Sherman et al., 2009). The reconstruction shows the *T* = 12 icosahedral envelope of the virion, depicting different types of capsomers (Freiberg et al., 2008; Sherman et al., 2009). Using the two model structures of Gn and Gc, we sought to identify their organization within capsomers by means of cross-correlation and built a model for the entire glycoprotein layer of the virion.

**Figure 2:**
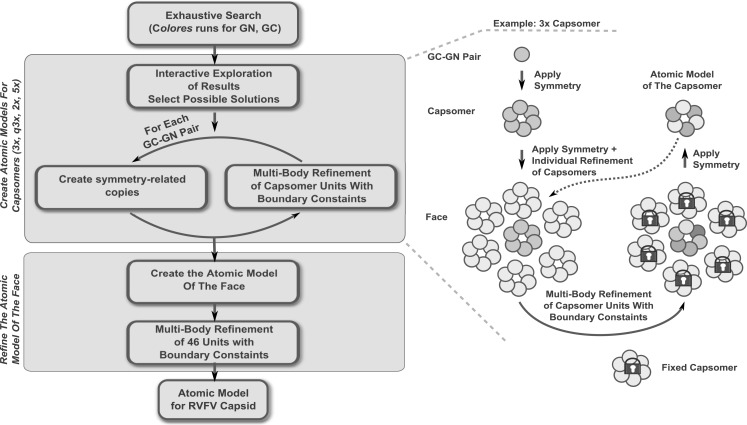
**Schematic representation of the modeling steps undertaken to create the atomic model of the RVFV envelope**. A detailed description of the individual steps is found in the text.

The glycoprotein layer is composed of capsomers showing different symmetry order (Freiberg et al., 2008; Huiskonen et al., 2009; Sherman et al., 2009). Pentons are located around the fivefold symmetry axis while hexons organize around the threefold, quasi threefold, and twofold axes. Although an icosahedral symmetry is imposed when reconstructing the cryoEM map of the virus, the hexons show different symmetry orders and can be averaged to increase the level of detail of the volumetric data. Such practice is common in modeling structures at low resolution, where averaging is applied to increase the signal-to-noise ratio of the data. First, the three different types of hexons were extracted, aligned, and then an averaged volume from the 11 copies was computed (rotations included). This averaged hexon, displaying a sixfold symmetry, was used to construct an average density for the asymmetric unit and the corresponding triangular face. The cryoEM density of the averaged face was utilized as target volume inside the envelope for the global docking of the Gc and Gn glycoproteins, respectively, inside the envelope. An exploration of all possible translations and rotations (9° step size) was performed for each glycoprotein with the colores tool of the Situs package (Wriggers, 2010). This exhaustive search allowed the estimation of the optimal cross-correlation coefficient, providing the list of top scoring placements. Colores also provided the optimal score and corresponding rotation for each voxel in the cryoEM map. This 3D scoring landscape was further investigated using interactive peak search, as described below. Due to the resolution of the cryoEM map, the top scoring placements provided by the exhaustive search were identified in the high-density regions of the map. Such arrangement of glycoproteins generated an atomic model with major steric clashes and prevented the assembly of the capsomers according to the Gn/Gc ratios estimated by Sherman et al. (2009). Therefore, we further investigated the results of the exhaustive search using interactive exploration techniques (Heyd and Birmanns, 2009) provided by the molecular modeling software Sculptor (Birmanns et al., 2011). This approach permitted us to augment the selection of cross-correlation peaks with expert knowledge such as the Gn/Gc ratio inside the capsomers. Multiple docking locations were thus selected for each type of glycoprotein resulting in several Gn/Gc pairs considered for further modeling steps. Each Gn/Gc pair was subjected to the procedure described in Figure 2.

First, the interactively selected placements were employed to create an initial model of the hexon located at the threefold axis. This atomic model, composed of 6xGc and 6xGn units, was also placed in the neighboring capsomers. We proceeded with a multi-body Powell refinement analysis of the raw volume of these fragments (as described in Birmanns et al., 2011), while at the same time applying boundary constraints. Such a local optimization simultaneously refines the translation and rotation of each glycoprotein in the capsomer by maximizing the cross-correlation coefficient. As multiple fragments are considered at the same time, the refinement prevents the glycoproteins from overlapping or from causing major steric clashes. The technique permits the introduction of boundary constraints in the form of atomic models describing the neighboring capsomers. Such constraints were not well defined in the first steps of the modeling and therefore the individual glycoproteins building the neighboring capsomers were also considered in the multi-body refinement. As the different types of capsomers were identified, the neighboring capsomers became available and were utilized as constraints in the refinement. No symmetry was technically considered during the refinement, yet the units effectively adopted the symmetry exhibited by the capsomer volume. For example, a threefold symmetry became apparent when refining the B capsomers which are organized around the threefold symmetry axis. The multi-body refinement was iterated several times until the placement of the glycoproteins was stable. As an atomic model was generated for each type of capsomer, a final multi-body refinement was undertaken to create the asymmetric unit. Forty-six units, 23 Gc and 23 Gn glycoproteins, were simultaneously refined while constraining the 15 neighboring capsomers.

### Intra- and inter-capsomer placement of RVFV Gn and Gc

**Figure 3:**
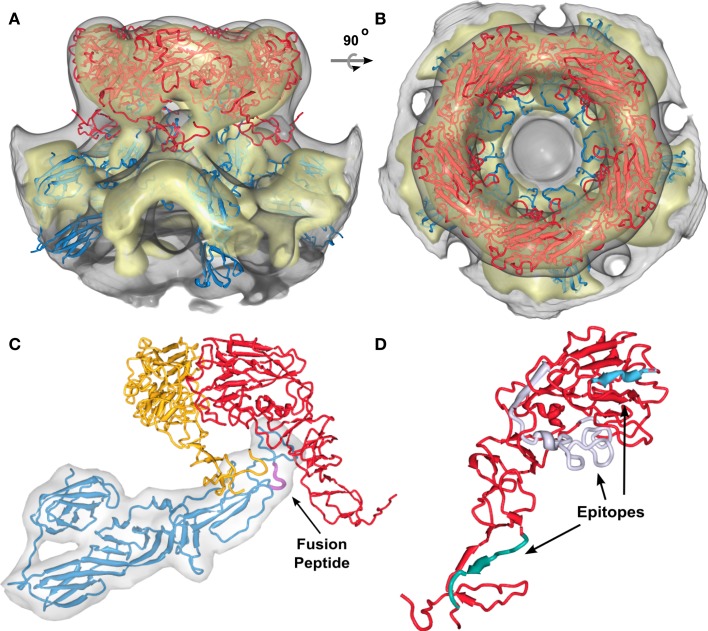
**Positioning of the Gn and Gc molecular models into the RVFV cryoEM reconstruction for the top scoring model**. **(A,B)** Show the glycoprotein arrangement within a penton extracted from the cryoEM density. The cryoEM density is represented as a gray transparent capsomer and the glycoprotein monomer models are indicated in red (Gn) and blue (Gc). Gn could only be positioned in the outer caldera of the capsomer and Gc in the skirt region of the capsomer. Two different viewing angles are shown (side-view, and top-view). **(C)** One structural unit (Gn-Gc heterodimer) and an adjacent Gn monomer have been extracted from the docking results shown in **(A)**. Within the basic structural unit, the head domain of the Gn model (red and yellow) covers domain II of Gc. The predicted location of the fusion peptide shown in domain II of Gc is highlighted in magenta and indicated by the black arrow. **(D)** Epitopes for three monoclonal antibodies recognizing Gn (Keegan and Collett, 1986) are highlighted. These epitopes are corresponding to the monoclonal antibodies 4-32-8D (gray), 4-D4 (blue), and 3C-10 (green).

**Figure 4:**
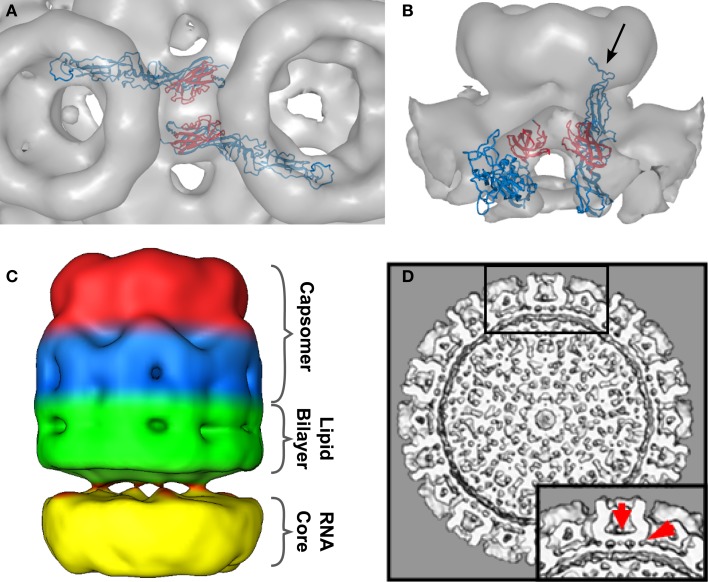
**Intercapsomer connections for the top scoring model**. **(A)** Top-view of two neighboring capsomers (gray cryoEM density) with two Gc monomers shown in blue. The domain III’s (red) are very well positioned within the ridges connecting adjacent capsomers. The fusion peptide is directed to the capsomer center. **(B)** Side-view of one capsomer along the tunnel located beneath the connecting ridges. Two Gcs are shown and their proposed position within the cryoEM density. The black arrow indicates the location of the fusion peptide within domain II. The domain IIIs are highlighted in red to indicate their placement within the ridges. **(C)** CryoEM density of one extracted penton at a very low threshold (0.54). The outer region of the capsomer is indicated in red (representing mainly Gn molecules), the capsomer base in blue (representing mainly Gc molecules), the lipid envelope in green, and the density corresponding to the RNP core is shown in yellow. Densities spanning the gap between the lipid bilayer and the RNP core are representing the glycoprotein cytoplasmic tails. **(D)** Surface-shaded representation of the central section of the RVFV cryoEM map viewed along the fivefold orientation. The sections show glycoprotein protrusions on virus surface, lipid bilayer, and RNP core. In the lower right corner a blow-up of the boxed area is shown. Red arrows point to clearly defined densities spanning the lipid bilayer. These densities represent glycoprotein transmembrane domains and are located either on the outer edge of the capsomer or directly beneath the connecting channels.

**Figure A1:**
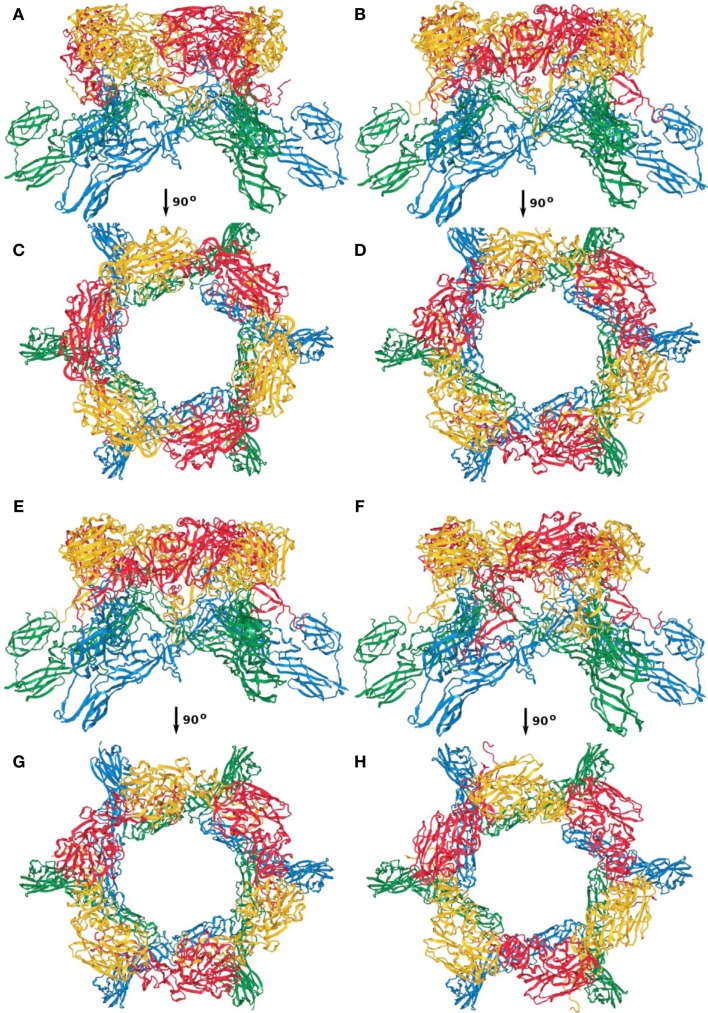
**Capsomer twofold axis; (A,C) first; (B,D) second; (E,G) third; (F,H) fourth top-scoring model**.

**Figure A2:**
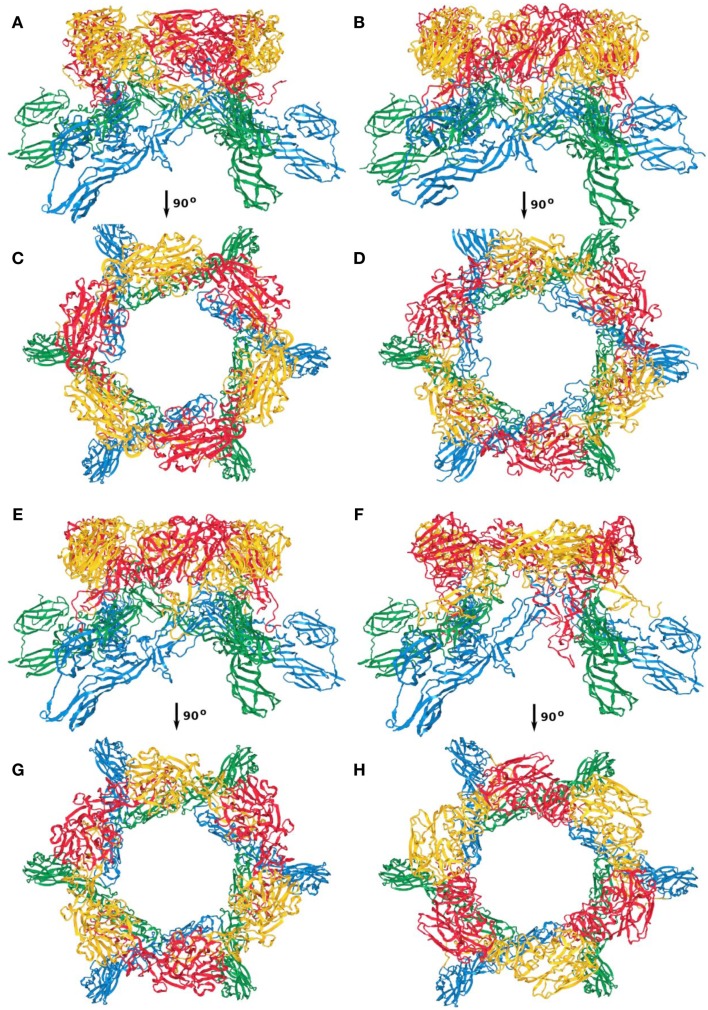
**Capsomer threefold axis; (A,C) first; (B,D) second; (E,G) third; (F,H) fourth top-scoring model**.

**Figure A3:**
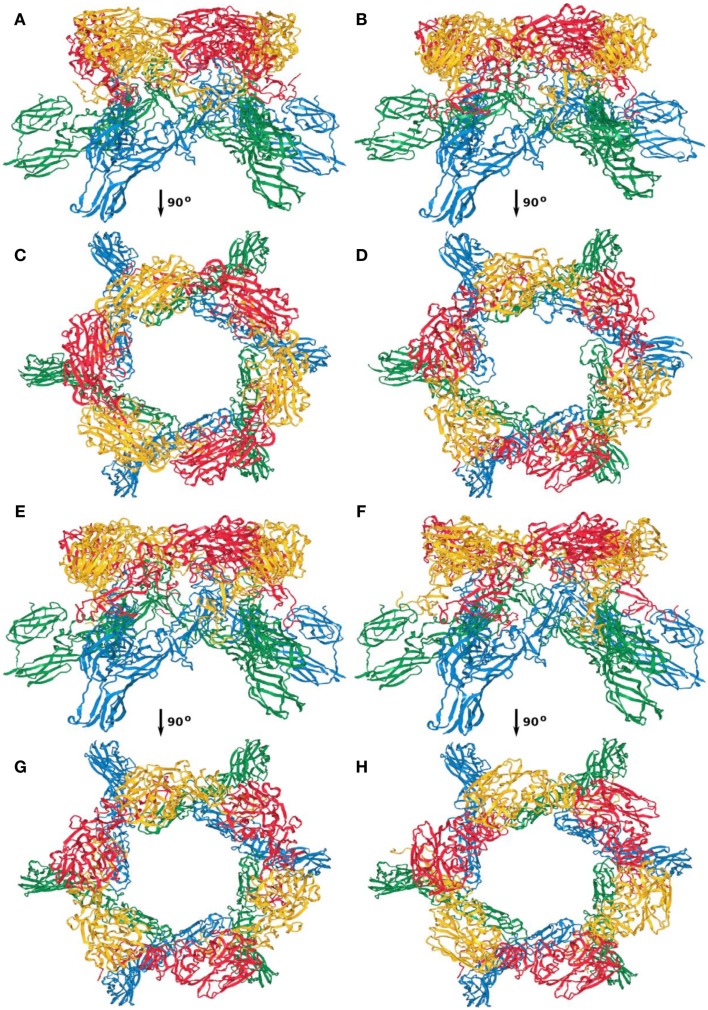
**Capsomer quasi-threefold axis; (A,C) first; (B,D) second; (E,G) third; (F,H) fourth top-scoring model**.

**Figure A4:**
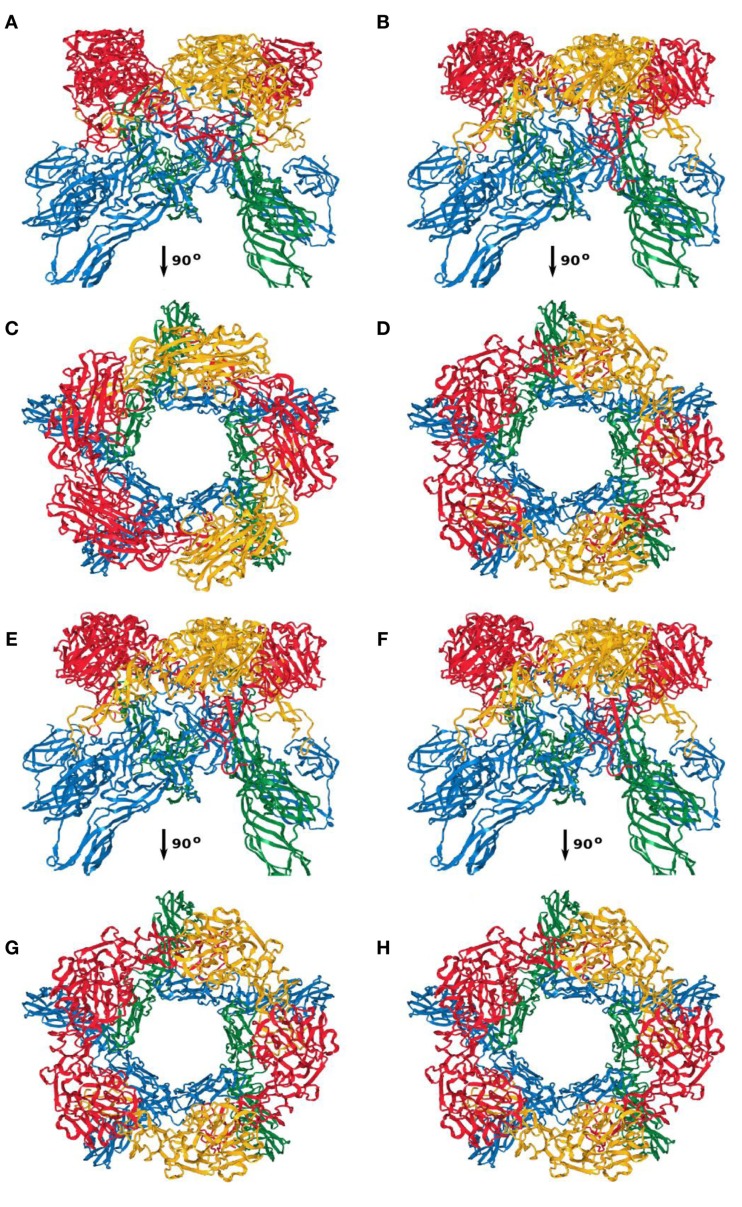
**Capsomer fivefold axis; (A,C) first; (B,D) second; (E,G) third; (F,H) fourth top-scoring model**.

We applied the described procedure (Figure 2) to 11xGn/Gc pairs obtained by combining the interactively selected Gc and Gn glycoproteins. Some of these pairs were discarded during the modeling as it become apparent that they prevented the generation of models with good stereochemical quality and appropriate Gn/Gc ratios. At the end of the procedure, four models were produced with cross-correlation coefficients above 0.783 (Figures A1–A4 in Appendix). The top scoring model had a correlation of 0.798 and is shown in Figures 3 and 4. This model had an estimated volume of approx. 1,300,000 Å^3^ for the hexon and approximately 1,100,000 Å^3^ for the pentons, in agreement with our previous calculations (Sherman et al., 2009).

Although the resolution of the 3D map of RVFV was limited, we were able to derive an assembly model through docking of the molecular Gn and Gc models using an iterative refinement and neighboring constraints (Figures 3A–C). In total, four possible arrangements of the glycoproteins in the virion envelope were identified and the predicted arrangement of the two glycoproteins leads to both, homo- and hetero-dimeric contacts between Gn and Gc (Figures A1–A4 in Appendix).

While our approach generated four possible models for the virion envelope, the organization of the glycoproteins is conserved between these models (Figures A1–A4 in Appendix). The Gc glycoprotein forms the icosahedral scaffold and remains consistent in the four models. It can be ascribed to the density identified as the viral “skirt” around the base of each capsomer. On the other hand, the Gn glycoprotein is placed in the protruding envelope yet has different angles relative to the scaffold. A close investigation of possible placements of Gn allowed us to group our four models into two main classes, in which Gn has a mirrored orientation with roughly ±45° relative to the scaffold (Figures A1–A4 in Appendix). The two possible placements are a result of the overall fold of the Gn glycoprotein as derived from homology modeling. The large globular domain of Gn drives the glycoprotein in the protruding capsomer, however the C- and N-terminus form a stalk region of reduced dimension, that provides insufficient constraints for the registration and thus the two different orientations. Moreover, the Gn model is incomplete at the C-terminus due to the lack in similarity with known protein structures (which prevented a homology based modeling of the region). Current on-going research in the laboratory is focused on providing experimental data to differentiate between the two potential orientations of Gn reported on the sequence similarity between the Gn proteins from two bunyavirus genera, namely hantaviruses and tospoviruses, with the Sindbis virus E2 protein. However, no significant sequence similarity was detected between the phlebovirus Gn and alphavirus E2 proteins. This might explain why comparison of the structural model for RVFV Gn with the recently solved alphavirus E2 protein structure (Li et al., 2010; Voss et al., 2010) did not reveal any structural similarity. The location of the two RVFV glycoproteins suggested in our model is plausible as Gn fits into the outer density of the capsomers and the model is consistent with the available biological data on RVFV. Keegan and Collett (1986) localized distinct antigenic determinants on the Gn glycoprotein and we chose three of these mapped epitopes and highlighted them in our molecular model for Gn (Figure 3D). Two of these epitopes, which are recognized by neutralizing monoclonal antibodies, are surface exposed (highlighted in blue and gray in Figure 3D). The epitope recognized by a non-neutralizing and non-protective antibody is located within the predicted stem region of Gn (highlighted in green in Figure 3D). In our model for Gn, this region interacts with domain II of Gc and also covers the fusion loop (highlighted in Figure 3C). The placement of Gc within the RVFV particle has similarities to that of the alphavirus E2 arrangement (Roussel et al., 2006). Domain II of E2 is the main interacting domain with E1, E2 has a position within the spike with a slight upward orientation on the virion surface and also forms the skirt of the spike (Li et al., 2010; Voss et al., 2010).

In the cryoEM reconstructions of RVFV, a strong density bridging neighboring capsomers has been described (Freiberg et al., 2008; Huiskonen et al., 2009; Sherman et al., 2009). These ridges are located halfway between the rim of the capsomer and the lipid bilayer of the virion. Inside these ridges a channel approximately 18 Å in diameter runs between adjacent capsomers and interconnects the inner cavities of the neighboring capsomers. In our model of the glycoprotein arrangement, Gc can be placed into the dense region of these ridges (Figure 4A). Specifically, the domain III of two Gc molecules from adjacent capsomers filled the density (highlighted in red in Figures 4A,B). In the side-view of the structure, one can clearly see how domain III forms the tunnel-like structure (Figure 4B). Further, the position of the fusion peptide oriented to the capsomer center is displayed (arrow in Figure 4B).

A similar model for the RVFV envelope was also obtained when building the Gc glycoprotein structure based on that of the Chikungunya virus E1 protein (Voss et al., 2010; data not shown). Again, Gn forms the protrusion spikes of the capsomers, while Gc is the main component of the icosahedral scaffold. Similarly, the domain III of Gc is the main component of the ridges between the capsomers. However, in this model the stem-like region of Gn is partially involved in the formation of the ridges as well (data not shown). Unlike in the previous model, in this model the fusion peptide located within Gc, points more outward from the capsomer but is still covered by the Gn glycoprotein.

## Discussion

The family *Bunyaviridae*, the largest RNA virus family with more than 350 named isolates, is organized into five genera based upon genetic and antigenic differences (Elliott, 2009). While many studies have focused on molecular aspects of transcription, replication, pathogenesis, and vaccine development, little is known about the structural organization and physical interactions of bunyavirus glycoproteins within the virion. Recently, cryoEM structures have been solved for the phleboviruses RVFV (Freiberg et al., 2008; Huiskonen et al., 2009; Sherman et al., 2009) and Uukuniemi virus (Overby et al., 2008), and the hantaviruses Tula (Huiskonen et al., 2010) and Hantaan viruses (Battisti et al., 2011). These structures did not only increase our basic knowledge regarding the assembly of the member viruses of this important virus family but also revealed that the bunyavirus glycoproteins can occur in multiple arrangements. While phlebovirus glycoproteins are arranged on the virion surface in *T* = 12 icosahedral symmetry, the hantavirus glycoproteins are arranged in a grid-like pattern. It is possible that the size of the glycoprotein molecules and the number of their TMD are factors contributing to the different arrangement of the glycoproteins on the surface of the member viruses of the various genera. However, due to the lack of an experimentally proven structure for any entire bunyavirus glycoprotein, we applied fold recognition structure prediction to generate 3D structural models for the RVFV Gn and Gc ectodomain monomers. The glycoprotein structures have been further analyzed in combination with the RVFV cryoEM structure previously solved by our group and others. Identifying the organization of the glycoproteins in the cryoEM envelope was achieved by using a modeling framework involving global and constrained local search. This framework was developed for RVFV, yet it may be applied to other multi-component assemblies.

### Hypothetical assembly model for rift valley fever virus

The two RVFV glycoproteins, Gn and Gc, are organized in 122 distinct capsomers on the virion surface, extending ~96 Å above the lipid envelope. Our docking framework (Figure 2) allowed the identification of four potential arrangements of the glycoproteins Gn and Gc within the virion envelope (Figures A1–A4 in Appendix). These models are mainly intended to represent a starting point for future research in analyzing the overall architecture of the phlebovirus envelope, as well as the virion assembly and fusion process. While we are aware of the fact that the described interactions between Gn and Gc homology models cannot be used to draw detailed conclusions at the molecular level, we can make the statement that Gn-Gc heterodimers form the basic structural unit in the capsomers in each of our four models. We hypothesize that hexons and pentons are comprised of six and five Gn-Gc heterodimers, respectively, with Gn being more solvent exposed and forming the capsomer spike and the Gc protein lying partially underneath, closer to the lipid membrane and forming the capsomer base. This arrangement is likely, since neutralizing monoclonal antibodies against both Gn and Gc have been described (Besselaar and Blackburn, 1991). In addition to interactions between Gn and Gc within each heterodimer, there are also interactions between neighboring structural units. A Gc molecule from one heterodimer contacts the stalk region of an adjacent Gn molecule, which is part of the neighboring heterodimer (Figure 3C). A recent study has shown that hantavirus glycoproteins form complex intra- and inter-molecular disulfide bonds between Gn and Gc, which contributes to the assembly and stability of the virus particle (Hepojoki et al., 2010). The RVFV Gn and Gc ectodomains used for our molecular modeling have 23 and 20 cysteines, respectively, and it is possible that similar inter- and intra-molecular disulfide bonds are present as well.

For our generated molecular models, we found significant structural matches between the RVFV Gn and the receptor binding domain of the Influenza virus hemagglutinin protein, and a separate match between the RVFV Gc protein and the alphavirus E1 protein. Since earlier bioinformatic investigation of the bunyavirus Gc protein has already predicted it to be a class II viral fusion protein (Garry and Garry, 2004), our findings for RVFV Gc were expected.

The alphavirus spike complex consists of a trimer of heterodimers [(E1-E2)_3_] and is mediated by interactions between E2 and E1 TMDs (Lescar et al., 2001; Pletnev et al., 2001). Even though we did not include the glycoprotein TMD and CTD in our fold predictions, it is possible that the Gn and Gc proteins interact with each other via their transmembrane regions and that the glycoproteins interact with the ribonucleoprotein complex via their Gn/Gc cytoplasmic tails. The interaction of the TMDs may represent an additional determinant in the heterodimer assembly. This hypothesis is strengthened by our description of protein densities spanning the space between the RNP core and the lipid bilayer within the RVFV particle (Sherman et al., 2009; Figure 4C). A recently published study by Piper et al. (2011) described the requirement of the RVFV Gn protein for genome packaging and showed that the Gn cytoplasmic tail is necessary for this process. In our RVFV cryoEM reconstruction we noticed the presence of densities spanning the virus envelope at the positions of capsomers (Sherman et al., 2009). These densities most likely represent the Gn and Gc TMDs and seem to be situated directly at the center of the ridges between neighboring capsomers and at the outer edges of the capsomers (red arrows in Figure 4D).

In contrast to many other lipid enveloped RNA viruses, bunyaviruses do not contain a matrix protein that has the function of linking and stabilizing the nucleocapsid and viral envelope proteins. Based on our model, we suggest that a highly organized arrangement of the Gn and Gc glycoprotein ectodomains is responsible for overall virion stability and that the capsomer–capsomer interactions play a central role in defining the icosahedral virion symmetry.

Multiple monoclonal antibodies against RVFV Gn and Gc have been described (Besselaar and Blackburn, 1991, 1994) and the epitopes on the ectodomain of Gn have been mapped (Keegan and Collett, 1986). In our model, the epitopes for the monoclonal antibodies 4-D4 and 4-32-8D, which have neutralizing and protective functions, are localized and surface-exposed in the globular head domain of Gn (Figure 3D). This domain caps Gc domain II and fusion loop and it may be that the neutralizing effect of these two antibodies is explained by either preventing receptor binding or potential rearrangement of Gn post-receptor attachment and, hence, inhibition of fusion, since the fusion loop will not be exposed to the host membrane. The epitope recognized by another monoclonal antibody, 3C-10, which has been described as non-neutralizing and non-protective in the mouse model has been localized in the stalk region of the Gn model (Figure 3D). In our model, this region can be found to be localized close to the ridges, connecting adjacent capsomers (Figure 3A). It is possible that this epitope is not freely accessible in the native conformation within the virion. The Gc domain III forming the capsomer connections may represent a steric block preventing antibody binding.

**Figure 5:**
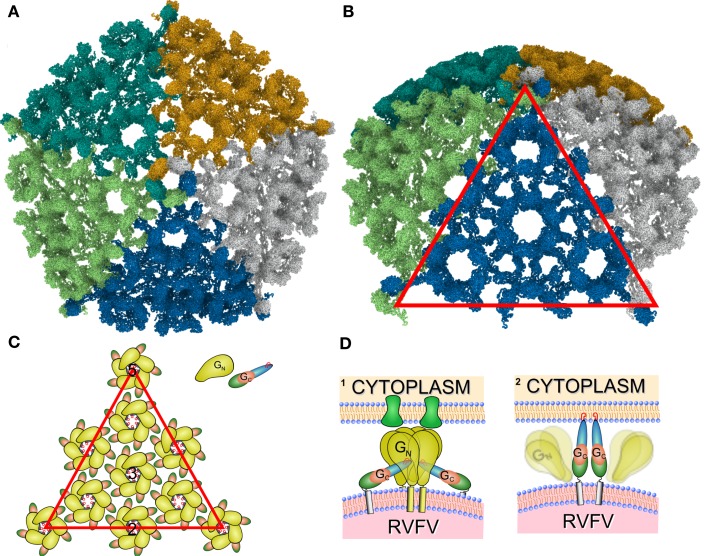
**Overview of the RVFV glycoprotein shell**. **(A)** The proposed *T* = 12 icosahedral protein layer formed by Gn and Gc. Individual subunits are color coded. **(B)** Tilted representation as shown in **(A)**. The red triangle represents one triangular face. **(C)** Schematic representation of the Gn and Gc contacts. Drawn is one of the 20 triangular faces of the icosahedrons enclosing the RVFV particle and the distribution of the Gn and Gc glycoproteins [corresponding to red triangle in **(B)**]. Black numbers denote icosahedral two-, three-, and fivefold symmetry axes. Gn monomers are represented as bulb-like structures in yellow, and Gc monomers as a tube-like structure. The individual domains are represented in red (domain I), blue (domain II), and green (domain III). The fusion peptides are indicated as red circles, and are pointing to the capsomer center. **(D)** Hypothetical model of the RVFV – host cell interaction. The RVFV glycoproteins Gn and Gc are represented according to our model and show similarities to the alphavirus E1 and E2 proteins. (1) Gn is depicted as the receptor binding protein and binds to the host cell receptor (green). (2) After receptor binding the uptake of the RVFV particle is initiated and an acidification step of the endocytic vesicle triggers the dissociation of Gn and Gc. This results in the formation of potentially Gc trimers (in accordance with current models for class II fusion proteins) and insertion of the fusion peptides into the host cell membrane.

In conclusion, structural models have been developed for the RVFV glycoproteins, Gn and Gc. The structural aspects of these protein models allowed us to generate four putative assembly models indicating how Gn and Gc may interact within and between capsomers. The top scoring model (as indicated by the highest cross-correlation coefficient) for the icosahedral shell of RVFV is presented in Figure 5. Our model has certain similarity to the described assembly model of alphaviruses, in terms of the fact that in bunyavirus surface proteins the receptor binding and membrane fusion activities most likely reside in two different glycoproteins (similar to the E1 and E2 glycoproteins in alphaviruses). However, while the alphavirus spike is formed by trimers of E1/E2 heterodimers, RVFV Gn/Gc heterodimers are organized in pentameric and hexameric capsomers. In flaviviruses, the E protein is responsible for both receptor binding and fusion. Further, the fusion peptide of the RVFV Gc protein sticks up and is oriented against Gn, similar to the findings for the alphavirus E1 and E2 proteins, whereas in the flaviviruses the fusion peptides are held down and are oriented against the interface of the E protein domain I and III.

The presented arrangement of Gn and Gc and description of their interactions may play an important role in glycoprotein folding and maturation, capsomer and virus assembly, virus fusion, and neutralization of infection. On-going site-directed mutagenesis experiments using a reverse-genetics system (Ikegami et al., 2005) are currently being used to evaluate the proposed glycoprotein interactions. The new information reported in this study, will not only impact our understanding of the assembly of phleboviruses and other bunyaviruses, but may also be exploited in furthering our understanding of the complex antigenic interactions of the many member viruses of the family *Bunyaviridae*. Such structural studies are hoped also to contribute to the design of effective antivirals.

## Conflict of Interest Statement

The authors declare that the research was conducted in the absence of any commercial or financial relationships that could be construed as a potential conflict of interest.

## Footnotes

^1^http://www.compbio.dundee.ac.uk/www-jpred/

^2^http://expasy.org/

^3^http://www.enzim.hu/hmmtop/

^4^http://bp.nuap.nagoya-u.ac.jp/sosui/

^5^http://www.cbs.dtu.dk/services/TMHMM/

^6^http://www.cbs.dtu.dk/services/NetNGlyc/

^7^http://meta.bioinfo.pl/submit_wizard.pl

^8^http://ffas.ljcrf.edu/ffas-cgi/cgi/ffas.pl

^9^http://salilab.org/modeller/

^10^http://situs.biomachina.org/

^11^http://sculptor.biomachina.org/

## Appendix

### Capsomer architecture

Between the four models, the overall architecture is preserved, with RVFV Gc forming the scaffold of the capsomers (blue and green ribbon representation in Figures A1–A4) and RVFV Gn being localized in the protruding envelope (red and yellow ribbon representation in Figures A1–A4). A close investigation of the different models revealed that the angle of the Gn monomers relative to the scaffold is different between the four models. The cross correlation coefficient was estimated for each model relative to the entire envelope. In order to construct the model of the entire envelope, 60 copies of the asymmetric unit were placed according to the icosahedral symmetry. The cross correlation coefficients were 0.798, 0.790, 0.785, and 0.783, for the first, second, third, and fourth top-scoring model.
